# A Fully Transparent Resistive Memory for Harsh Environments

**DOI:** 10.1038/srep15087

**Published:** 2015-10-12

**Authors:** Po-Kang Yang, Chih-Hsiang Ho, Der-Hsien Lien, José Ramón Durán Retamal, Chen-Fang Kang, Kuan-Ming Chen, Teng-Han Huang, Yueh-Chung Yu, Chih-I Wu, Jr-Hau He

**Affiliations:** 1 Computer, Electrical and Mathematical Sciences and Engineering (CEMSE) Division, King Abdullah University of Science & Technology (KAUST), Thuwal 23955-6900, Saudi Arabia; 2 Department of Electrical and Computer Engineering, Purdue University, West Lafayette, Indiana 47907, USA; 3 Institute of Photonics and Optoelectronics & Department of Electrical Engineering, National Taiwan University, Taipei 10617, Taiwan, ROC; 4 Institute of Physics, Academia Sinica, Taipei 11529, Taiwan, ROC

## Abstract

A fully transparent resistive memory (TRRAM) based on Hafnium oxide (HfO_2_) with excellent transparency, resistive switching capability, and environmental stability is demonstrated. The retention time measured at 85 °C is over 3 × 10^4^ sec, and no significant degradation is observed in 130 cycling test. Compared with ZnO TRRAM, HfO_2_ TRRAM shows reliable performance under harsh conditions, such as high oxygen partial pressure, high moisture (relative humidity = 90% at 85 °C), corrosive agent exposure, and proton irradiation. Moreover, HfO_2_ TRRAM fabricated in cross-bar array structures manifests the feasibility of future high density memory applications. These findings not only pave the way for future TRRAM design, but also demonstrate the promising applicability of HfO_2_ TRRAM for harsh environments.

With the recent advances in nanotechnology, there is an increasing interest in harsh electronics. Specifically, there are two driving forces. First is the increasing requirement from oil, gas, aircraft, aerospace, nuclear and military industry, which require devices to operate in extremes of radiation, pressure, temperature and chemically corrosive environments^1^. The second driving force is the rise of transparent electronics. Different from conventional electronics, transparent electronics employing low cost, transparent and flexible substrates, enables a wide range of new applications, such as artificial skins^2^, free-form displays^3^, flexible solar cells^4^, smart clothes^5^, and sensor implants^6^. However, the direct exposure of transparent devices to the outside ambience makes them susceptible to corrosive, erosive, and high-temperature environments, limiting their practical applications. Therefore, it is of great urgency to develop and optimize reliability and durability of transparent electronics for harsh environment applications. Particularly, memory devices indispensable for any kind of electronic systems draw most of attentions.

Resistive random access memory (RRAM) is considered as one of most promising candidates for next generation memory due to its excellent capability and feasibility to be implanted on different substrates such as papers, soft plastic, and non-planar substrates^7^,^8^. Among several kinds of RRAMs, ZnO-based RRAMs could be highly noteworthy because of its high speed^9^, low power consumption^10^, superior scalability^11^, and multi-functionality toward transparent electronics^12^. However, the resistive switching characteristics of ZnO-based RRAMs are strongly influenced by the ambiences, including interfacial oxygen chemisorption^13^, moisture^14^, and atmospheric corrosion^15^. As a result, the major hindrance for practical applications of ZnO RRAM is the non-uniform memory switching due to the pronounced surface effect^16^. Much work has been reported recently in dealing with these issues. For instance, Yang *et al.* reported that the ambient effects on transparent RRAM (TRRAM) can be remarkably suppressed by introducing graphene electrodes as a surface passivation layer, which eliminates the detrimental effect of chemisorbed oxygen molecules^17^. Huang *et al.* observed that the incorporation of fluorine into ZnO surfaces can effectively restrain the surface effect and improve the resistive switching characteristics of ZnO-based RRAM^18^. Nevertheless, these methodologies focus only on either modifying or engineering metal oxide surfaces rather than intrinsic properties of metal oxide materials. For providing long-term device reliability, a more efficient way is to find/fabricate/modify the metal oxide material as inert as possible to suppress the surface effects.

HfO_2_, recognized as the most stable and reliable candidate in the field of RRAM has been widely investigated in several aspects, such as high density memory architecture^19^, nanosecond switching capability^20^, high temperature stability^21^, and neuromorphic computation system^22^. Compared with ZnO, HfO_2_ exhibits not only relative inertness to the ambient oxygen adsorption, but also comparable transparent nature, which can be beneficial for the development of future TRRAM to operate under harsh conditions^23^. However, toward practical applications of utilizing TRRAMs for future harsh environments, a critical issue is to understand their device durability and switching uniformity under various kinds of harsh conditions in addition to the high temperature, and fewer reports can be found currently to reach relevant results.

In this study, a sandwiched structure of indium-tin oxide/hafnium oxide/indium-tin oxide (ITO/HfO_2_/ITO) fabricated at room temperature for TRRAM is demonstrated, which exhibits average transmittance of 77.64% within the visible wavelength region from 400 to 800 nm. The ON/OFF ratio, defined as the high resistance state (R_H_) over the low resistance state (R_L_), is approximately 15 can be obtained for HfO_2_ TRRAM, and no significant degradation can be observed for more than 100 cycles within cycling endurance test. The retention time measured at 85 °C is 3 × 10^4^ sec. The statistical analysis including cell-to-cell and device-to-device tests for over 100 cells are conducted, verifying the excellent switching uniformity of HfO_2_ TRRAM. Moreover, little fluctuations in switching parameters of HfO_2_ TRRAM can be perceived under various oxygen partial pressure, moisture, radiation and corrosive agent exposure, validating its outstanding durability in contrast to ZnO TRRAM. Furthermore, the HfO_2_ TRRAM is fabricated into the cross-bar array configuration, confirming its feasibility for future high-density memory applications. This work demonstrates a comprehensive investigation of utilizing a highly potential HfO_2_ TRRAM for harsh environment applications, offering not only excellent environmental stability against various ambiences, but also high density compatibility toward future transparent electronics.

## Results

### Optical property and binding energy characterization

**Figure 1:**
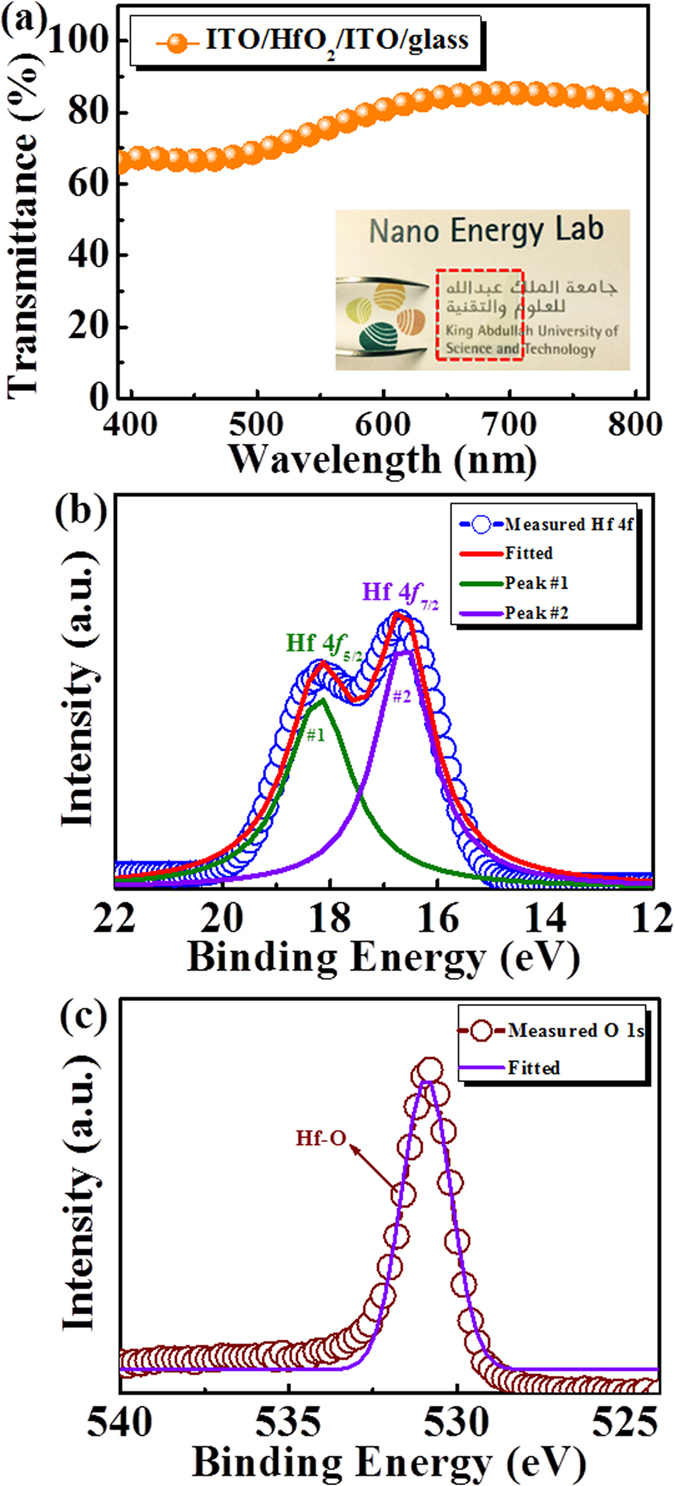
(**a**) The transmittance spectrum of the sandwiched structure ITO/HfO_2_/ITO within the visible region from 400 to 800 nm. The inset shows the as-fabricated device. The background can be observed through the device without any refraction or distortion. (**b**) Hf 4f and (**c**) O 1 s XPS spectra of as-deposited HfO_2_ film. The hollow sphere is the measured data of HfO_2_ and the solid line is the fitting result.

To quantitatively examine transparency, the transmittance spectrum of the as-fabricated structure ITO/HfO_2_/ITO/glass was investigated, as shown in Fig. 1(a). The average transmittance of the ITO/HfO_2_/ITO/glass is 77.64% within the visible wavelength region from 400 to 800 nm. The photograph of the ITO/HfO_2_/ITO/glass is marked in a dashed-line rectangle in the inset of Fig. 1(a). The “King Abdullah University of Science and Technology-Nano Energy Lab” logo beneath the device can be perceived clearly due to the optical transparency of the device.

The chemical composition of the HfO_2_ thin film was characterized by an X-ray photoelectron spectroscopy (XPS) at room temperature. As shown in Fig. 1(b,c), the peaks located at 18.4 and 16.7 eV can be referred to the binding energies of Hf 4 f_5/2_ and 4 f_7/2_ orbitals, respectively. In addition, the peak located at 530.2 eV is related to the standard O 1 s orbital. These results confirm the formation of HfO_2_ by sputtering technique^21^.

### Resistive switching characteristics

**Figure 2:**
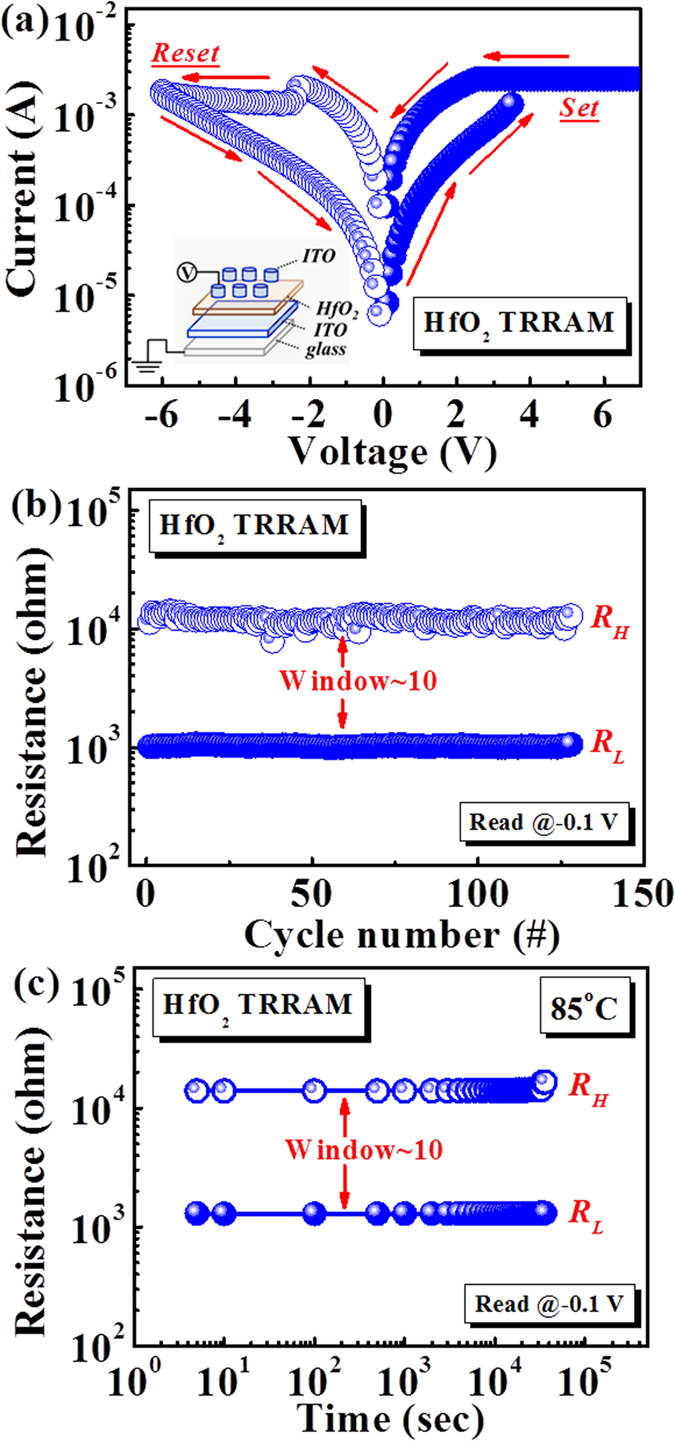
(**a**) Typical *I*–*V* characteristics of HfO_2_ TRRAM under atmospheric condition. The corresponding configuration of two-terminal devices is depicted in the inset of (**a**). (**b**) Endurance, and (**c**) Retention characteristics of HfO_2_ TRRAM at 85 °C. R_L_ and R_H_ were read at −0.1 V for 3 × 10^4^ sec, and no significant degradation is observed.

Figure 2 shows the typical resistive switching characteristics of HfO_2_ TRRAM, including current-voltage (*I–V*) characteristics, endurance, and retention test at 85 °C. For comparison, a control sample with the structure of ITO/ZnO/ITO (ZnO TRRAM) was also prepared. During the measurements, a DC voltage was applied on the top electrode while the bottom electrode was grounded. Current compliance, imposed for the forming processes to prevent permanent destruction of dielectric thin films, was set to 1 μA. The forming voltage is approximately 9 V. After the forming process, a bipolar switching characteristic can be obtained. As shown in Fig. 2(a), the device is initially situated in R_H_. By sweeping the voltage above a positive threshold value, a sudden increase in current is observed (as denoted by the arrow of **Set**) indicating that the device is switched to the low resistance state. Then, an abrupt drop of current occurs when the voltage decreases below a negative threshold value (as denoted by the arrow of **Reset**), which indicates that the device switches back to the high resistance state. These results demonstrate the reversible and steady bipolar switching characteristics of HfO_2_ TRRAM. (Description of the schematic)

To evaluate the reliability of HfO_2_ TRRAM, endurance, and retention properties were measured. Figure 2(b) shows the endurance property for 130 successive resistive switching cycles. The resistance values were read at −0.1 V in each DC sweep. It is clear that the ON/OFF ratio is larger than 10 within 130 switching cycles and no conspicuous decay can be observed in both resistance states. The two well-resolved distributions of resistance in the two states ensure a sufficient and clear window for read operation. These results indicate that the switching characteristics of HfO_2_ TRRAM are reproducible and stable. Figure 2(c) shows the retention property of HfO_2_ TRRAM at 85 °C. It is clear that, for both states, the resistance can be maintained over 3 × 10^4^ sec, demonstrating the excellent non-volatility of HfO_2_ TRRAM.

### Effect of oxygen adsorption

**Figure 3:**
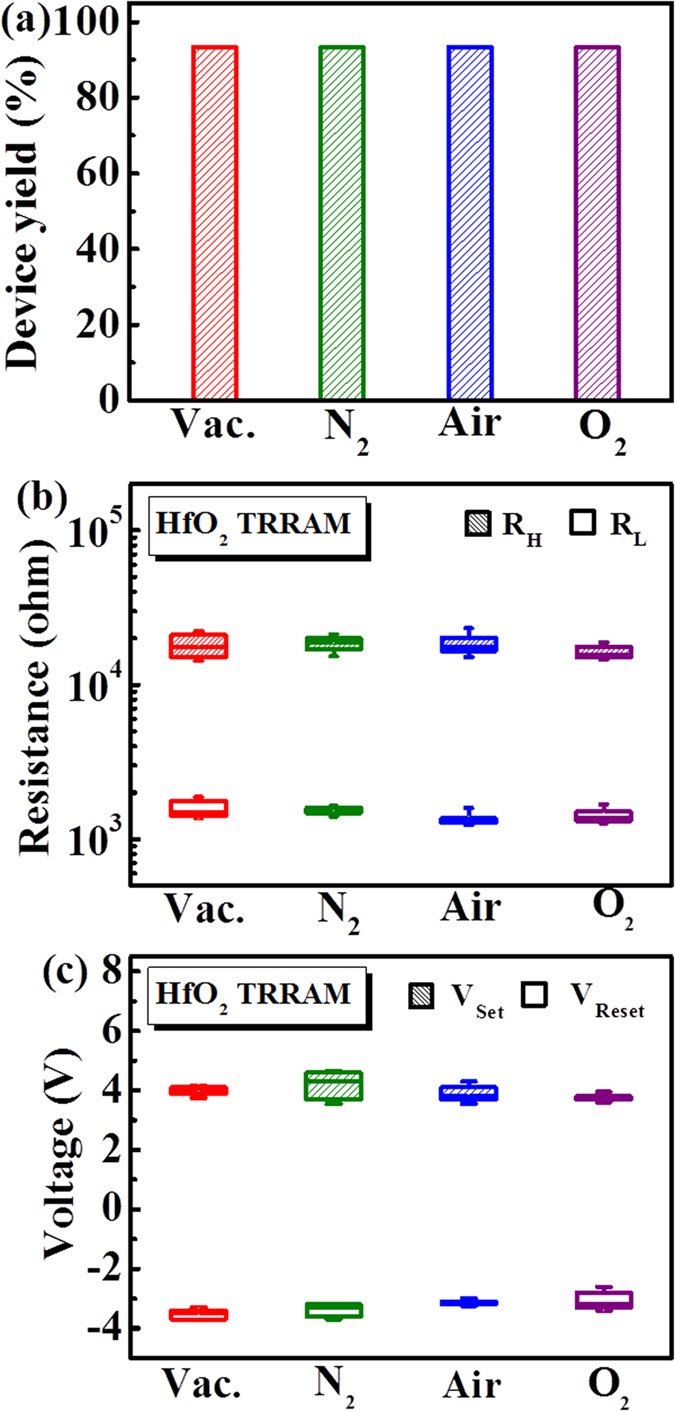
Resistive switching characteristics of HfO_2_ TRRAM under ambient conditions of vacuum (Vac.), nitrogen (N_2_), air, and oxygen (O_2_). (**a**) Device switching yield, (**b**) R_H_ and R_L_ distributions, and (**c**) V_set_ and V_reset_ distributions.

Next, the durability of HfO_2_ TRRAM for harsh environments is explored. It is well-known that oxygen adsorption at surface of metal oxides act as electron traps for charge carriers, which results in the increase of surface potential and deterioration of device performance^16^,^17^,^18^,^24^,^25^,^26^,^27^,^28^. Hence, the effect of oxygen partial pressure on performance of HfO_2_ TRRAM is first examined. We performed endurance test of 100 cycles for each cell under four different ambient conditions (vacuum, N_2_, air, and O_2_), simulating environments with low, medium and high oxygen concentrations. A statistical analysis for over 100 cells is conducted for evaluating switching yields, resistance distributions (R_H_ and R_L_) and switching voltage distributions (V_Set_ and V_Reset_), as shown in Fig. 3(a–c), respectively. As it can be seen, the percentage of switching yield, defined as the ratio of the amount of cells exhibiting resistive switching characteristics over 100 cycles without any Set or Reset failure to the amount of total cells, is quite high (>90%) and uniform for all the ambient conditions. Moreover, the resistance states and switching voltages remain fairly stable with negligible deviation under all the ambiences. The results show the insensitive properties of HfO_2_ TRRAM toward oxygen adsorption, indicating that the detrimental surface effects on resistive switching characteristics can be suppressed by using HfO_2_ as a replacement of ZnO. In fact, HfO_2_ conventionally serves as a surface passivation layer on ZnO-based transistors and diodes due to its chemical stability and inertness^23^,^29^.

### Effect of moisture adsorption

To get further insight into the environmental influence on resistive switching characteristics of metal oxides, a damp-heat (DH) treatment conducted at 85 °C and 90% relative humidity (RH) was implemented to study the effect of moisture adsorption^30^. As shown in Fig. S1(a),(b) in the Supplementary Information, HfO_2_ and ZnO TRRAMs exhibit distinct switching characteristics during the DH treatment. For ZnO TRRAM, the resistance in both states was greatly degraded, and the two states were indistinguishable after 12-hour treatment (*i.e.*, device failure). In contrast, the two distinct memory states of HfO_2_ TRRAM remain stable and uniform after 100-hour DH treatment. The DH test results validate that HfO_2_ TRRAM is more sustainable than ZnO TRRAM in both humidified and high-temperature environments due to its superior chemical stability.

### Effect of atmospheric corrosion

Moreover, the corrosion robustness of ZnO and HfO_2_ TRRAMs were investigated under formic acid exposure, as shown in Fig. S1(c),(d) in the Supplementary Information. Though the ZnO-based devices have exhibit−ed excellent performances in the field of electronics and optoelectronics, the atmospheric corrosion due to Zn^2+^ dissociated from the surface remains a significant issue^15^,^31^,^32^. When exposed to an acidic environment, the ON/OFF ratio of ZnO TRRAM decreases significantly with exposure time, and fails after 2100-min, as shown in Fig. S1(c) in the Supplementary Information. The resistance degradation of R_H_ can be attributed to the adsorption of formic acid molecule which causes the dissociation of Zn^2+^ near the surface and the decrease in thickness shown in Fig. S2(a) I–IV in the Supplementary Information. In contrast, the effect of atmospheric corrosion on HfO_2_ TRRAM is remarkably eliminated, and the resistance exhibits little dependence on acid exposure, as shown in Fig. S1(d) in the Supplementary Information. After 6000-min acid exposure, the window between R_H_ and R_L_ remains clear, demonstrating the superior corrosion robustness of HfO_2_ TRRAM to acid solutions. These results are supported by the negligible decrease in thickness and roughness of the HfO_2_ thin films after formic acid exposure, as shown in Fig. S2(a) V–VIII in the Supplementary Information. Figure S2(b),(c) in the Supplementary Information also present that the transmittance spectra of HfO_2_ remain constant with exposure time, while the transmittance of ZnO increases from 71.56% to 91.38% at 400 nm. The increase of transmittance can be attributed to the deterioration of ZnO film thickness and roughness during formic acid exposure, which can correspond to the device failure of ZnO TRRAM after 300 min in Fig. S1(c) in the Supplementary Information. Meanwhile, it has been reported that the etching rates of HfO_2_ thin fim are extremely low in formic acid, sulfuric acid, and oxalic acid solutions^33^. With the excellent corrosion robustness, HfO_2_ thin films have been widely used as a surface passivation layer in microelectromechanical systems^34^.

### Effect of proton irradiation

The other important environmental factor that might cause device damage is proton irradiation^35^,^36^. Long-term exposure under proton irradiation can cause shifts of *I*–*V* characteristics, larger leakage current, high power consumption, and malfunction of electronic devices. In general, these degradations are related to the interaction between proton-induced charges and bulk defects, where oxide and interface traps are usuallly created^37^. To investigate the radiation tolerance, the as-fabricated TRRAMs were irradiated with 2 MeV protons, where proton fluences range from 10^11^ to 10^16^  cm^−2^. Note that the protons with the energy less than 2 MeV and the fluences ranging from 10^1^ cm^−2^ to 10^8^  cm^–2^ occupy a region about 1*L*-2*L* above earth’s surface, where *L* is approximately equal to the geocentric distance of a field line in the geomagnetic equator^38^. The resistance distributions of ZnO and HfO_2_ TRRAMs under the impact of various proton fluences are also shown in Fig. S1(e),(f) in the Supplementary Information. For ZnO TRRAM, apparent fluctuations in R_H_ and R_L_ can be observed. Conversely, the resistance distributions of HfO_2_ TRRAM are congruent, showing reliable switching characteristics under proton irradiation. These results suggest that HfO_2_ TRRAM is more reliable than ZnO TRRAM in highly-radiative environments.

Specifically, variations in switching parameters of HfO_2_ TRRAM are relatively lower than ZnO TRRAM after proton irradiation, which may correlate to the difference in radiation-hardness of ZnO and HfO_2_. Previously, it has been reported that radiation hardenss of metal oxides are closely related to ionic binding strength^39^. In addition, proton irradiation damge which primarliy comes from fomation of radiation-induced defects has also been reported to associate with binding energies in metal oxides^40^. It is likely that, for metal oxides, the higher the binding strength, the better the radiation hardeness. Meanwhile, it is well understood that HfO_2_ possess higher bonding strength than ZnO, which may further imply better radiation hardness of HfO_2_. More experiments are currently conducted for clarifying the mechanism and will published elsewhere. While the precise mechanism cannot be determined, it is reasonable to state that HfO_2_ TRRAM is a promising candidate for future transparent memory devices to operate under extremely radiative environments.

### ON/OFF ratio

**Figure 4:**
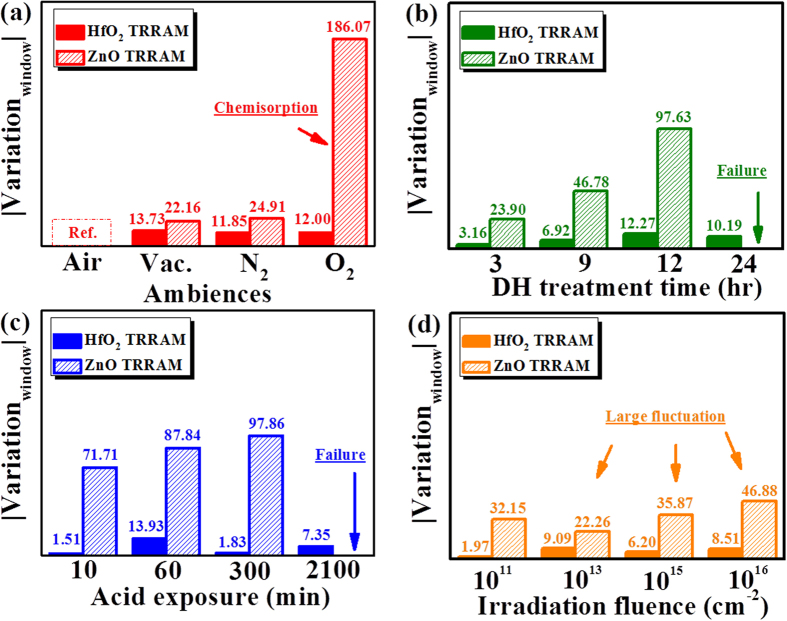
Variations on ON/OFF ratio as a function of (**a**) oxygen partial pressure (**b**) moisture treatment, (**c**) acid exposure, and (**d**) proton irradiation. Air in (**a**) means that the ZnO and HfO_2_ TRRAMs are measured under the ambience of air, without moisture treatment, acid exposure, and proton irradiation.

Toward practical RRAM application, maintaining stable ON/OFF ratio of memory devices under various environmnets is one of the key issues that needs to be addressed. Herein, we evaluate the variation of ON/OFF ratio of ZnO and HfO_2_ TRRAMs under a variety of environments (including different oxygen partial pressure, moisture, acid exposure, and proton irradiation) by a simple equation below.


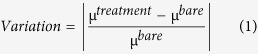


where μ^treatment^ and μ^bare^ represent the average value of ON/OFF ratio obtained from treated (treatment) and untreated (bare) devices, respectively, after 100 cycling tests. Bare condition means that the ON/OFF ratio of TRRAMs are measured under the ambience of air without moisture treatment, acid exposure, or proton irradiation. In Fig. 4(a–d), it can be found that the ON/OFF ratio of ZnO TRRAM strongly depends on the environmental conditions. Deterioration and fluctuation of ZnO TRRAM significantly increase with the oxygen concentration, humidity, formic acid, and proton irradiation fluences. Conversely, little variation in the ON/OFF ratio of HfO_2_ TRRAM (less than 15%) is observed for diverse environmental conditions. In short, the as-fabricated HfO_2_ TRRAM exhibits not only high resistance to oxygen chemisorption, but also excellent durability under moisture, acid exposure, and proton irradiation.

**Figure 5:**
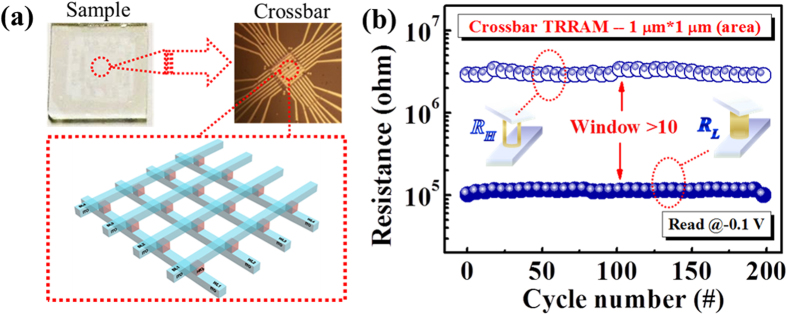
(**a**) A schematic of cross-bar configuration of HfO_2_ TRRAM. Image of the as-fabricated device is shown on the upper left. The device area of each TRRAM cell inside the cross-bar array is 1 μm^2^. (**b**) Endurance characteristics of HfO_2_ TRRAM cell in cross-bar array.

To access the applicability of HfO_2_ TRRAM for future memory technology, a cross-bar array configuration of HfO_2_ TRRAM is illustrated in Fig. 5(a). The optical image of the as-fabricated HfO_2_ TRRAM in the cross-bar array in the (upper-right inset) is the enlargement from the central area of the as-fabricated sample (upper left inset) in Fig. 5(a). Note that the packing density of RRAM in cross-bar array can be 1000-times higher than that of current static random-access memory cells^41^. The endurance property of HfO_2_ TRRAM cell in cross-bar array was measured and presented in Fig. 5(b). Little fluctuations can be observed for more than 100 cycles, indicating the feasibility of HfO_2_ TRRAM for future high-density memory applications.

## Discussion

Stacked layers of ITO/HfO_2_/ITO deposited at room-temperature are demonstrated as a TRRAM for harsh environment applications. The HfO_2_ TRRAM exhibits an average transmittance of 77.64% in the visible range (from 400 nm to 800 nm), and reliable resistive switching characteristics. To investigate effects of the harsh conditions on resistive switching characteristics of TRRAMs, various ambient conditions, including high oxygen partial pressure, high moisture (relative humidity = 90% at 85 °C), and corrosive agent exposure were implemented. In comparison with ZnO TRRAM, HfO_2_ TRRAM shows outstanding tolerance and consistent switching characteristics against diverse ambiences. Moreover, HfO_2_ TRRAM exhibits superior immunity from proton irradiation (2 MeV with fluences up to 10^16^ cm^−2^), showing great potential to operate under extremely radiative environments. Furthermore, the cross-bar array fabricated with HfO_2_ TRRAM demonstrates the feasibility for future high-density memory applications in see-through electronics. These explorations give insights not only in realizing an environmentally stable and high-density compatible TRRAM, but also in developing practical applications of TRRAM for transparent electronic systems with high reliability requirements.

## Methods

### TRRAM Fabrication

A commercial glass substrate was pre-cleaned by alcohol and deionized water to avoid the contamination from the ambience. ITO thin film of 100 nm thickness as the bottom electrode was deposited on glass substrate by rf-sputtering technique. A HfO_2_ thin film with a thickness of 50 nm was deposited by rf-sputtering technique afterwards. Finally, as the top electrode of the device, a 100 nm thick ITO thin film with a diameter of 200 μm was deposited by sequential sputtering process with a metal shadow mask. Note that all the processes mentioned above were carried out at room temperature.

### Characterization

The transmission spectrum of the whole device was measured by (UV/visible V670). For radiation tolerance testing, the HfO_2_ TRRAM was irradiated at room temperature using a 2 MeV proton beam from a 3 MV tandem accelerator (NEC 9SDH-2, National Electrostatics Corporation). The typical current of the proton beam was 2–50 nA (the current increases with increasing fluences). with the beam fluences ranged from 10^11^ cm^−2^ to 10^16^ cm^−2^ at the sample target. Keithley 4200-SCS semiconductor characterization system was used to measure resistive switching characteristics of the as-fabricated HfO_2_ TRRAM. Field-emission transmission electron microscopy (JEOL JEM-7100F) was used to investigate the microstructures of ZnOand HfO_2_ thin films.

## Additional Information

**How to cite this article**: Yang, P.-K. *et al.* A Fully Transparent Resistive Memory for Harsh Environments. *Sci. Rep.* **5**, 15087; doi: 10.1038/srep15087 (2015).

## Supplementary Material

Supplementary Information
