# Supplementary material for: A Fully Transparent Resistive Memory for Harsh Environments
Source: Sci Rep. 2015 Oct 12;5:15087. doi: 10.1038/srep15087 (PMC4601025; doi:10.1038/srep15087)
Supplement: Supplementary Information [file srep15087-s1.pdf]

Supplementary Information for:

A Fully Transparent Resistive Memory for Harsh Environments

Po-Kang Yang[1,3], Chih-Hsiang Ho[2], Der-Hsien Lien[1], José Ramón Durán Retamal[1], Chen-Fang Kang[1,3], Kuan-Ming Chen[4], Teng-Han Huang[3], Yueh-Chung Yu[4], Chih-I Wu[3], and Jr-Hau He[1*]

[1]Computer, Electrical and Mathematical Sciences and Engineering (CEMSE) Division, King Abdullah University of Science & Technology (KAUST), Thuwal 23955-6900, Saudi Arabia.
[2]Department of Electrical and Computer Engineering, Purdue University, West Lafayette, Indiana 47907, USA.
[3]Institute of Photonics and Optoelectronics & Department of Electrical Engineering, National Taiwan University, Taipei 10617, Taiwan, ROC.
[4]Institute of Physics, Academia Sinica, Taipei 11529, Taiwan, ROC.
[*]E-mail: jrhau.he@kaust.edu.sa

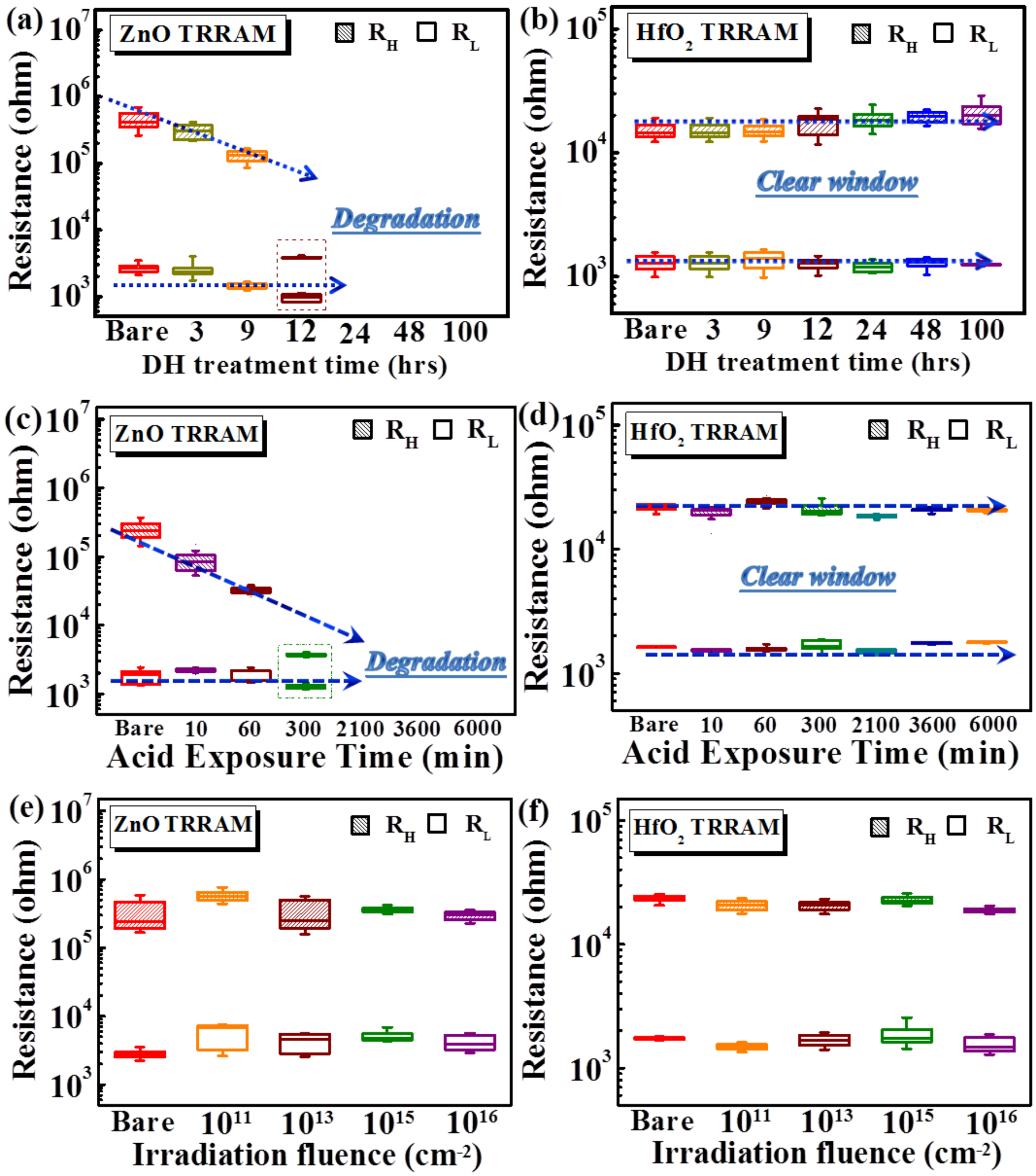

Figure S1. Resistance distributions of TRRAMs in harsh conditions. (a) The resistance distribution of ZnO TRRAM under damp heat tests for 100-hours with conditions (85 ℃, RH 90%). (b) The resistance distribution of HfO_2 TRRAM under damp heat tests for 100-hours with conditions (85 ℃, RH 90%). Resistance distributions in R_H and R_L of (c) ZnO TRRAM and (d) HfO_2 TRRAM under formic acid exposure. Resistance distributions in R_H and R_L of (e) ZnO and (f) HfO_2 TRRAM as a function of proton irradiation fluences.

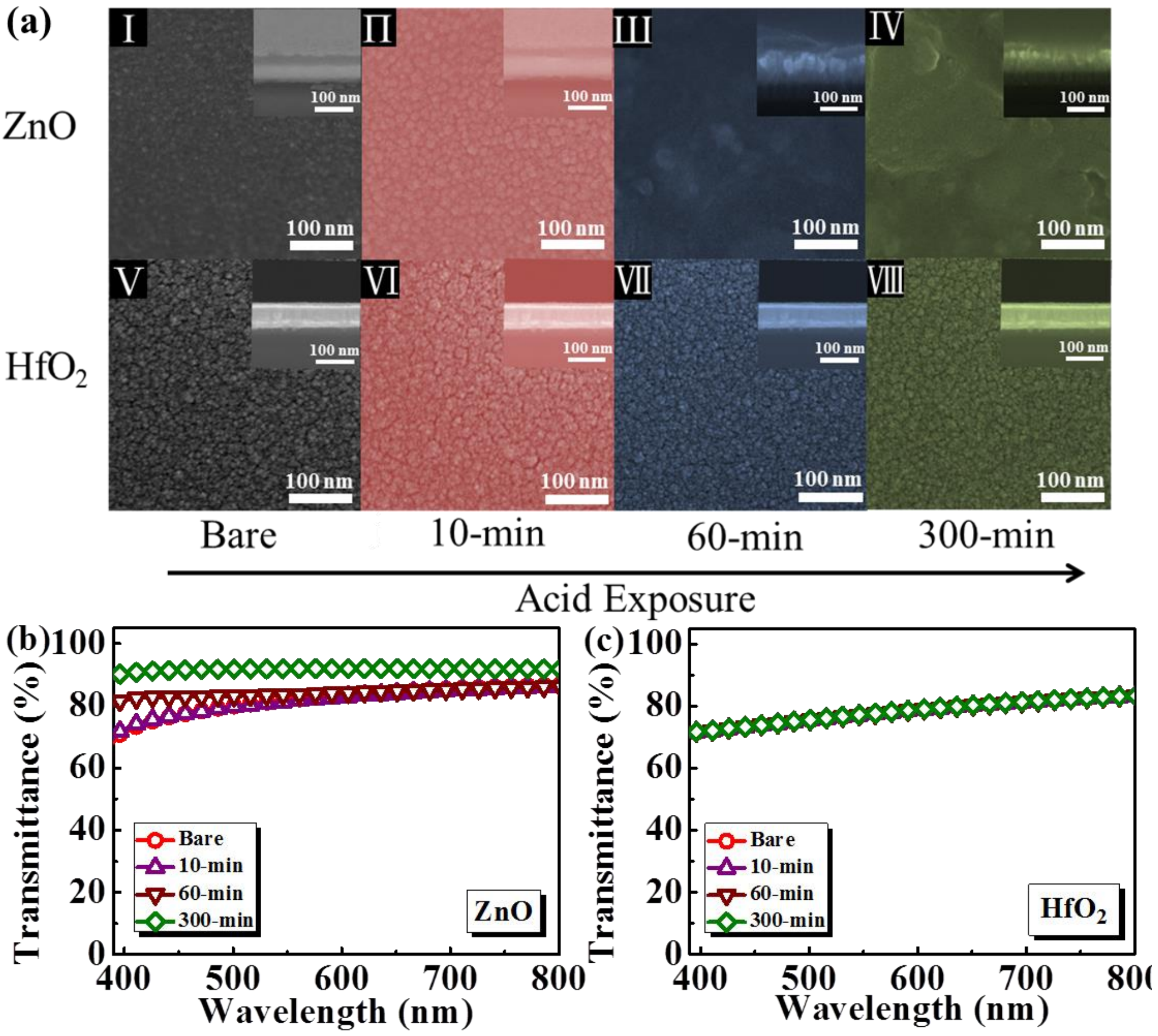

Figure S2. (a) Surface morphologies of (I) pristine ZnO and ZnO with (II) 10-min, (III) 60-min, (IV) 300-min, (V) pristine HfO_2 and HfO_2 with (VI) 10-min, (VII) 60-min, (VIII) 300-min formic acid exposure. The insets in (a) show the cross-sectional SEM images. Transmittance spectra of (b) ZnO and (c) HfO_2 thin films during formic acid exposure.
